# Establishment and application of the BRP prognosis model for idiopathic pulmonary fibrosis

**DOI:** 10.1186/s12967-023-04668-5

**Published:** 2023-11-11

**Authors:** Xiaoyun Cheng, Zhichao Feng, Boyu Pan, Qingxiang Liu, Yuanyuan Han, Lijun Zou, Pengfei Rong, Jie Meng

**Affiliations:** 1 Department of Pulmonary and Critical Care Medicine, The Third Xiangya Hospital of Central South University, Tongzipo Road 138, Yuelu District, Changsha, 410000 Hunan China; 2 Hunan Key Laboratory of Organ Fibrosis, Tongzipo Road 138, Yuelu District, Changsha, 410000 China; 3 Departments of Radiology, The Third Xiangya Hospital of Central South University, Tongzipo Road 138, Yuelu District, Changsha, 410000 Hunan China; 4 Departments of Orthopedics, The Third Xiangya Hospital of Central South University, Tongzipo Road 138, Yuelu District, Changsha, 410000 Hunan China

**Keywords:** Idiopathic pulmonary fibrosis, Pericardial adipose tissue, Pectoralis muscle, Lung function, Inflammation, Transplant-free survival

## Abstract

**Background:**

Idiopathic pulmonary fibrosis (IPF) is the most common idiopathic interstitial lung disease. Clinical models to accurately evaluate the prognosis of IPF are currently lacking. This study aimed to construct an easy-to-use and robust prediction model for transplant-free survival (TFS) of IPF based on clinical and radiological information.

**Methods:**

A multicenter prognostic study was conducted involving 166 IPF patients who were followed up for 3 years. The end point of follow-up was death or lung transplantation. Clinical information, lung function tests, and chest computed tomography (CT) scans were collected. Body composition quantification on CT was performed using 3D Slicer software. Risk factors in blood routine examination-radiology-pulmonary function (BRP) were identified by Cox regression and utilized to construct the “BRP Prognosis Model”. The performance of the BRP model and the gender-age-physiology variables (GAP) model was compared using time-ROC curves, calibration curves, and decision curve analysis (DCA). Furthermore, histopathology fibrosis scores in clinical specimens were compared between the different risk stratifications identified by the BRP model. The correlations among body composition, lung function, serum inflammatory factors, and profibrotic factors were analyzed.

**Results:**

Neutrophil percentage > 68.3%, pericardial adipose tissue (PAT) > 94.91 cm^3^, pectoralis muscle radiodensity (PMD) ≤ 36.24 HU, diffusing capacity of the lung for carbon monoxide/alveolar ventilation (DLCO/VA) ≤ 56.03%, and maximum vital capacity (VCmax) < 90.5% were identified as independent risk factors for poor TFS among patients with IPF. We constructed a BRP model, which showed superior accuracy, discrimination, and clinical practicability to the GAP model. Median TFS differed significantly among patients at different risk levels identified by the BRP model (low risk: TFS > 3 years; intermediate risk: TFS = 2–3 years; high risk: TFS ≈ 1 year). Patients with a high-risk stratification according to the BRP model had a higher fibrosis score on histopathology. Additionally, serum proinflammatory markers were positively correlated with visceral fat volume and infiltration.

**Conclusions:**

In this study, the BRP prognostic model of IPF was successfully constructed and validated. Compared with the commonly used GAP model, the BRP model had better performance and generalization with easily obtainable indicators. The BRP model is suitable for clinical promotion.

**Supplementary Information:**

The online version contains supplementary material available at 10.1186/s12967-023-04668-5.

## Background

Idiopathic pulmonary fibrosis (IPF) is a progressive and destructive fatal lung disease. There are over five million IPF patients worldwide, and both the morbidity and mortality rates of IPF are increasing [1]. IPF pathogenesis mainly involves chronic inflammation, abnormal wound healing, cell aging, endoplasmic reticulum stress, etc. [2]. IPF usually has poor curative effect, with a median survival time of 2–5 years [3]. The disease progression and prognosis of IPF are highly heterogeneous [4]. Therefore, predicting the survival of patients with IPF and choosing the timing of lung transplantation are challenges for clinicians. In 2012, Ley et al. [5] developed a multidimensional scoring and prognostic staging system for IPF called the gender-age-physiology variables (GAP) calculator and GAP index, which is currently the most commonly used tool to predict the prognosis of IPF. However, the use of the GAP calculator is limited due to its lack of accuracy and assessment of baseline variables such as biomarkers and radiological markers [6]. It is necessary to develop a new prediction model with higher accuracy to improve IPF disease management. Moreover, IPF remains an incurable disease to date [7], partly due to its complex and poorly understood etiology, meaning that new multidisciplinary perspectives and joint indicators may be needed to achieve breakthroughs.

Evidence suggests that inflammatory factors play an important role in the pathogenesis of IPF. Chronic inflammation leads to epithelial mesenchymal transformation and abnormal activation of alveolar epithelial cells, and the latter secrete mediators that induce fibroblast migration, proliferation, and transformation into myofibroblasts. Fibroblasts and myofibroblasts continue to secrete extracellular matrix. Cytokines are also released to enhance inflammation and eventually collectively lead to pulmonary fibrosis [8, 9]. However, at present, the efficacy of anti-inflammatory drugs in animal trials and clinical studies of IPF is not exact, and sometimes contradictory results are reported. In the absence of an inflammation-based model, it is difficult to predict clinical outcomes in patients with IPF who receive anti-inflammatory therapy (such as an interleukin [IL]-6 antagonist or corticosteroids) [10, 11], which may lead to irreversible loss of lung function. Developing an inflammation-based prognosis model for IPF is crucial for evaluating anti-inflammatory treatment decisions.

Inflammation has been found to be correlated with body composition in recent years. IPF is an age-related disease; with aging, ectopic adipose inflammation tends to worsen, resulting in increased visceral fat volume; skeletal muscle fat infiltration (known as myosteatosis); and increased proinflammatory macrophages, cytokines, chemokines and adipocytokines [12]. Pericardial adipose tissue (PAT) is an abundant source of proinflammatory mediators and has been shown to be positively correlated with proinflammatory molecules (IL-6, tumor necrosis factor-α [TNF-α], monocyte chemotactic protein-1, and cluster of differentiation-11c) and profibrotic markers (collagen, transforming growth factor beta [TGF-β], and matrix metalloproteinase-3) [13, 14]. Excessive PAT suggests poor prognosis in various cardiopulmonary diseases, such as COVID-19, chronic obstructive pulmonary disease (COPD), pulmonary hypertension, obstructive sleep apnea syndrome, coronary heart disease, heart failure, and lung transplant recipients [5, 15]. The most common cause of death among patients with IPF is chronic respiratory failure. Skeletal muscle atrophy is highly prevalent in patients with malnutrition or respiratory failure that requires mechanical ventilation [16]. A low skeletal muscle index and muscle cross-sectional area have been shown to be related to an increased risk of IPF mortality [17]. Low pectoralis muscle radiodensity (PMD) measured by computed tomography (CT) imaging indicates myosteatosis [18]. A low PMD is an important predictor of poor clinical outcomes in various chronic diseases [19]. PAT, PMD and pectoralis muscle cross-sectional area (PMA) can be accurately and quickly quantified using CT according to the thresholds of different tissues [20, 21]. Chest CT is a guideline-recommended and readily available clinical tool for all IPF patients [22, 23]. However, the clinical value of CT quantification of body composition in the prognosis of IPF is still unclear. The main objective of the study was to develop an inflammation-related predictive model for transplant-free survival (TFS) of IPF patients, which may provide a more accurate risk assessment and help physicians make more informed personalized management decisions. Additionally, this model may have potential value in studying the mechanisms of inflammation in IPF and monitoring and evaluating anti-inflammatory treatments in IPF patients.

## Methods

### Patient population

The inclusion criteria were as follows: IPF patients who first visited the Xiangya Hospital or the Third Xiangya Hospital of Central South University between January 2016 and October 2019; were diagnosed according to the established criteria of the American Thoracic Society/European Respiratory Society guidelines [22, 24]; and underwent routine blood tests, biochemistry, lung function tests (including ventilation and diffusion) and HRCT within seven days of the first clinical visit. Patients with the following conditions were excluded from this study: currently experiencing acute exacerbation of IPF (AE-IPF) or any other acute inflammation; expected survival less than 90 days due to the presence of malignant tumors, severe liver or kidney dysfunction, or abnormal hematopoietic function; use of immunosuppressive agents (including glucocorticoids, cyclosporine A, and tacrolimus) or antifibrotic drugs (including interferon, D-penicillamine, and colchicine); incomplete data; or loss to follow-up within the past three months.

**Fig. 1:**
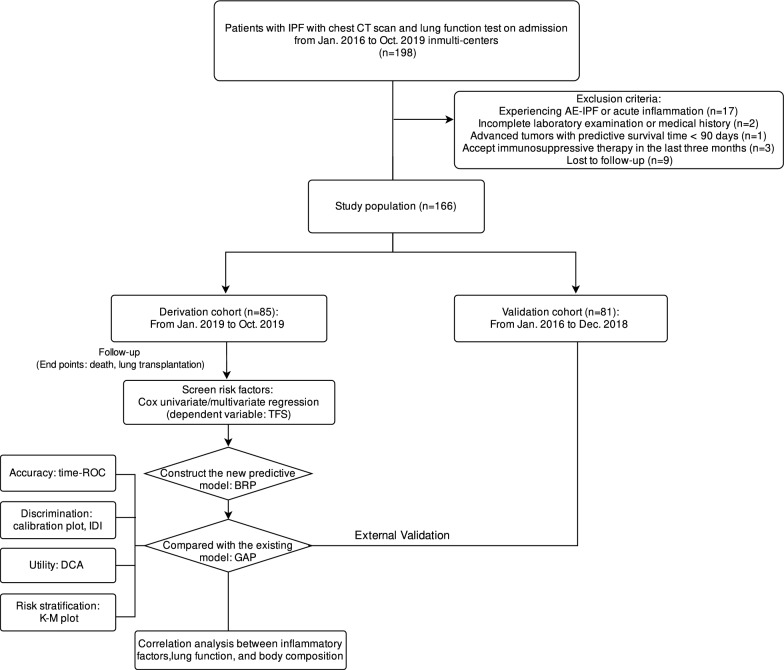
Flow chart

A total of 166 patients with IPF were ultimately enrolled. The training set was defined as 85 patients from the aforementioned two research centers from January to October 2019, and their early morning fasting peripheral blood samples were retained for the test of inflammatory factors. The validation set consisted of 81 patients from the two research centers from January 2016 to January 2019 (Fig. 1). Within the first six months after the initial diagnosis, all patients were followed up every 3 months. The follow-up frequency was every 6 months from 6 months to 3 years until the relevant outcomes of interest occurred. The primary outcome measure was the time from the first clinic visit to either death for any reason or lung transplantation. This study was approved by the Medical Ethics Committee of Xiangya Hospital (Code: 201812184), and all IPF patients signed informed consent forms.

### Measurement of pulmonary function, PAT, pectoralis muscle and inflammatory factors

Pulmonary function tests were conducted according to the guidelines of the American Thoracic Society/European Respiratory Society [25]. To avoid the influence of individual physical differences, pulmonary function indicators such as vital capacity (VC), forced expiratory volume (FEV), and diffusing capacity of the lung for carbon monoxide/alveolar ventilation (DLCO/VA) were expressed in the model as a percentage of predicted values (pred%) instead of absolute values. The DLCO/VA (pred%) values of 6 patients (6.98%) in the training set and 8 patients (9.88%) in the validation set could not be obtained, mainly due to poor lung ventilation function in these patients that made it impossible to perform diffusion function tests. To address this issue, DLCO/VA (pred%) was transformed into a categorical indicator, with those who could not undergo pulmonary diffusion function testing being defined as “unable to perform.” After the log-rank test, it was found that there was no significant difference in TFS between the “unable to perform” patients and those with DLCO/VA (pred%) ≤ 56.03; therefore, these patients were combined into one group.

**Fig. 2:**
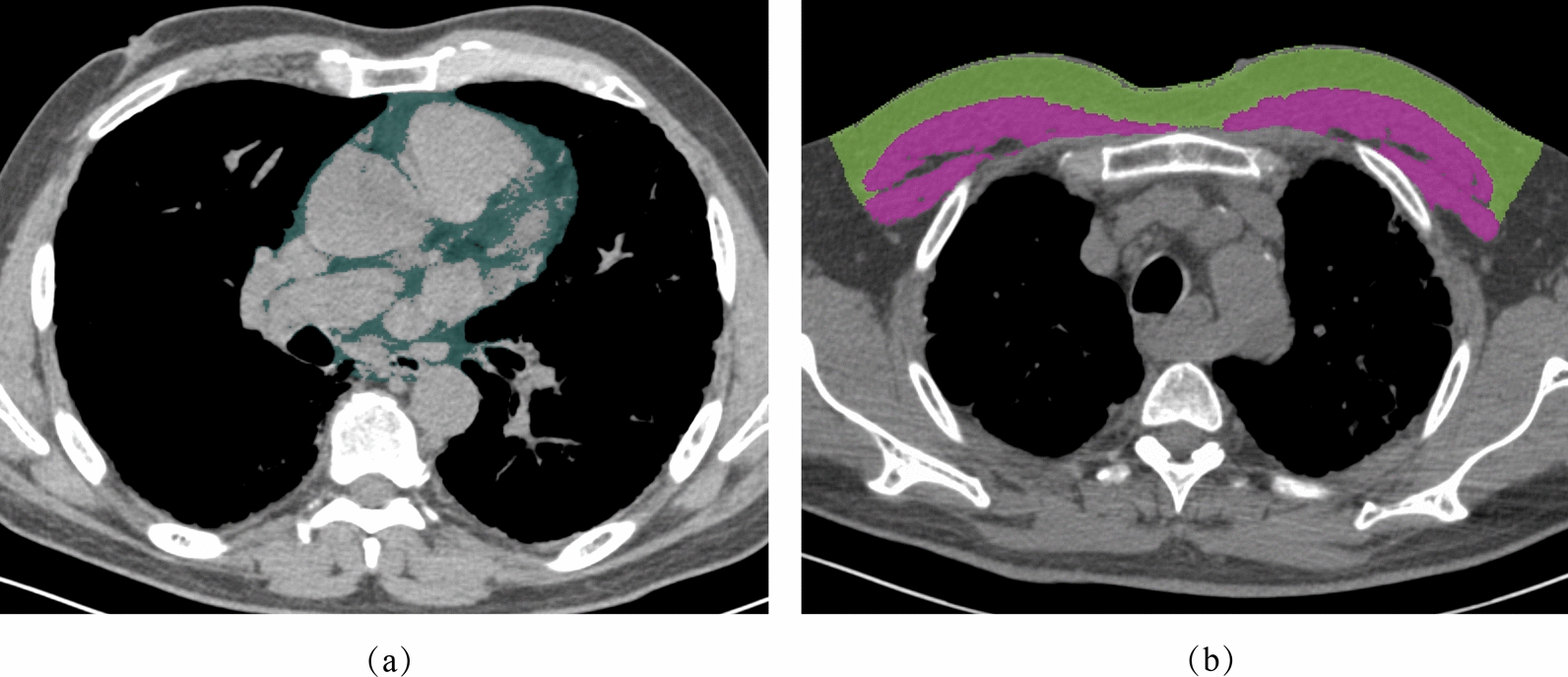
Representative CT images for the quantitation. **a** PAT (dark green); **b** pectoralis muscle (purple) and SAT of the chest wall (light green)

Quantitative analysis of body composition markers in the cross-sectional chest unenhanced CT examinations taken solely for the purpose of diagnosing IPF was performed using 3D Slicer software (version 4.10.2; https://www.slicer.org/). The area and attenuation of pectoralis muscle (including major and minor) and subcutaneous adipose tissue (SAT) were measured at the superior to the aortic arch level. SAT was defined as the region between the pectoralis muscle and the skin surface. Tissue Hounsfield unit (HU) thresholds were employed as follows: − 29 to 150 HU for PM and − 190 to − 30 for SAT. The PAT volume was also determined. PAT was defined as all tissue with the density of adipose tissue (– 190 to – 30 Hounsfield units [HU]) located between 15 mm above and 30 mm below the superior extent of the left coronary artery and bordered anteriorly by the chest wall and posteriorly by the aorta and bronchus. Measurements were manually performed by a single trained reader blinded to the outcomes (Z.F., a radiology research fellow with 10 years of experience). Representative images used for analyses are shown in Fig. 2.

Blood samples were centrifuged at 3000 rpm for 10 min, and serum was collected for measurement of the following cytokine levels using standard enzyme-linked immunosorbent assay techniques: human IL-1β (AiFang Biology, catalog number AF-03336H1), IL-4 (AiFang Biology, catalog number AF-03206H1), IL-6 (AiFang Biology, catalog number AF-03204H1), interferon-γ (IFN-γ, AiFang Biology, catalog number AF-03188H1), C-reactive protein (CRP, AiFang Biology, catalog number AF-03290H1), and TGF-β1 (AiFang Biology, catalog number AF-03245H1).

### GAP calculator and GAP index

According to the criteria established by Ley et al. [5] in 2012, the GAP calculator included sex, age, and two pulmonary function indicators, and points were assigned as follows: sex (0–1 points), with 1 point given for males and 0 points for females; age (0–2 points), with 0 points for ages 60 and above, 1 point for ages 61–65, and 2 points for ages above 65; FEV pred% (0–2 points), with 0 points given for > 75%, 1 point for 50–75%, and 2 points for ≤ 50%; and DLCO/VA pred% (0–3 points), with 0 points given for > 55%, 1 point for 36%-55%, and 2 or 3 points for ≤ 36% or inability to complete the test. Patients were classified according to the total score of the GAP calculator as follows: 0–3 points, GAP index I; 4–5 points, GAP index II; and 6–8 points, GAP index III.

### Survival assessment

The survival status of patients was confirmed by reviewing medical records or through telephone follow-up. If the patient had died, the study team obtained the information from authorized representatives. The definition of TFS was as described earlier. For patients who were still confirmed to be alive at 1100 days, the survival time was counted as 1100 days, and these patients were classified as censored cases in the analysis.

### Model development

Cox univariate and multivariate regression analyses were used to identify factors associated with TFS. Variables with a p value < 0.05 in the univariate regression analysis were entered in the Cox multivariate regression analysis. Each significant continuous variable in the Cox multivariate regression was transformed into a binary variable. Then, variables were screened through forward–backward stepwise regression to fit into the Cox proportional hazard model.

### Model comparison and internal/external validation

The newly developed blood routine examination-radiology-pulmonary function (BRP) score was compared with the GAP calculator in the training and validation sets. The accuracy of the two models was evaluated using the area under the time-dependent receiver operating characteristic curve (AUC). The discriminative ability of the models was assessed using calibration plots. The integrated discrimination improvement (IDI) value reflected the improvement of the BRP model over the GAP calculator. Internal validation was performed using bootstrapping (B = 500). The clinical utility of the models was evaluated using decision curve analysis (DCA). Finally, the patients were divided into three risk groups according to the BRP score at the 50th percentile (115 points) and 75th percentile (187 points) in the training set. Patients with a BRP score < 115 points had the lowest all-cause mortality or lung transplantation requirement, those with scores ≥ 115 and < 187 had intermediate risk, and those with scores ≥ 187 were in the highest risk group.

### Histopathology and immunohistochemistry

Lung tissues obtained from 10 patients with IPF, who underwent lung biopsies or lung transplant procedures, as well as from adjacent normal controls of 4 patients with lung cancer. These tissues were then embedded in paraffin and sectioned at a thickness of 4 μm. Hematoxylin and eosin (H and E) staining and Masson’s trichrome staining were performed for fibrosis score analyses [26]. The evaluation was performed blindly by two pathologists independently. For immunohistochemistry, the slides were incubated with the primary antibody against TGF-β1 (Proteintech, 21898-1-AP). The staining was analyzed using computerized morphometry (Image-Pro Plus 6.0 software, Media Cybernetics, Bethesda, MD, USA).

### Western blotting

Whole proteins from lung tissues of patients with IPF and normal controls were extracted, and western blot analysis was performed as described previously [27]. The primary antibody was a TGF-β1 antibody (Abcam, ab215715), and the secondary antibody was goat anti-rabbit IgG (CST, 7074S).

### Statistical analysis

Count data are presented as n (%) and were compared using the chi-square test. Continuous data are expressed as the median [25th percentile–75th percentile, Q1–Q3] and were analyzed using the Mann‒Whitney U test to assess homogeneity between the training and validation sets. The cutoff points of the variables were determined by the "surv_cutpoint" function of the "survminer" package. The Cox model was fitted and visualized using the "survival" package and "rms" package. Time-ROC and DCA were performed using the “timeROC” and “ggDCA” packages, respectively. Kaplan‒Meier survival curves and log-rank tests were used to compare the survival differences among the three groups. Spearman’s correlation analysis was used to investigate the correlations among serum inflammatory factors, imaging markers, and lung function in patients with IPF. In addition, histopathological scores of different risk stratifications according to the BRP model were compared by one-way analysis of variance. R Studio (version 4.2.0; R Studio, Boston, Massachusetts) was used for statistical analysis and graphing. All statistical tests were two-tailed, with p < 0.05 considered significant.

## Results

### Baseline characteristics of study participants

Table 1 summarizes the baseline clinical characteristics, treatment, pulmonary function, and body composition measurements of all enrolled patients. Of the 166 patients with IPF, the median age was 66.50 [57.00, 72.00] years, including 131 (78.9%) males and 35 (21.1%) females. The median body mass index (BMI) was 23.80 [22.10, 25.90] kg/m^2^. The most common comorbidities were COPD (39, 23.5%), hypertension (34, 20.5%), coronary heart disease (29, 17.5%), and diabetes (28, 16.9%). The presence of malignant tumors in patients with IPF appears to be not rare (14, 8.4%). In terms of laboratory tests, the median percentages of peripheral blood cell classifications were within the normal laboratory reference ranges. The oxygenation index was 357.00 [276.25, 407.00], indicating that more than a quarter of the patients had respiratory failure. VCmax and FEV1/FVC values indicated normal to mild obstructive ventilatory dysfunction. Sixty-one (36.75%) patients had a DLCO/VA ≤ 56.03 (pred %), indicating moderate to severe pulmonary diffusion dysfunction in over one-third of the patients in this study; among them, 6 patients (6.98%) in the training set and 8 patients (9.88%) in the validation set were unable to complete the pulmonary diffusion function test. Overall, the results of the study were consistent with the pulmonary function characteristics of IPF patients. Table 1 also presents the CT quantitation results for PAT, PMA, PMD, and subcutaneous adipose tissue area (SATA) and density (SATD). In terms of clinical treatment, half of the patients (83, 50.0%) had long-term use of pirfenidone, while approximately one-third (59, 35.5%) had long-term use of only N-acetylcysteine. Three patients in the training set and two patients in the validation set underwent lung transplantation surgery. The cumulative mortality rates at 1 year, 2 years, and 3 years for all enrolled patients were 21.1% (35/166), 39.8% (66/166), and 48.2% (80/166), respectively. Comparing the baseline characteristics between the training set and the validation set, there were no statistically significant differences in sex; BMI; or pectoralis muscle, pericardial, or subcutaneous adipose tissue CT quantitative results, suggesting a similar distribution of patient characteristics between the two sets. In comparison to the training set, the validation set had older patients (69.00 [61.00, 73.00] vs. 64.00 [57.00, 70.00] years, p = 0.042), a higher prevalence of combined coronary heart disease (24.7% vs. 10.6%, p = 0.029), and lower serum albumin levels (36.50 [33.00, 38.60] vs. 38.90 [35.10, 41.60] g/L, p = 0.001), indicating that the validation results of this study have certain extrapolation validity.

**Table 1:** The baseline data on demographics, auxiliary examinations, and CT quantification of body composition

Characters	Total (n = 166)	Training (n = 85)	Validation (n = 81)	p value
TFS (day)	1100.00 [421.25, 1100.00]	1008.00 [448.00, 1100.00]	1100.00 [417.00, 1100.00]	0.435
Death, n (%)	82 (49.4)	43 (50.6)	39 (48.1)	0.874
Male, n (%)	131 (78.9)	68 (80.0)	63 (77.8)	0.872
Age (years)	66.50 [57.00, 72.00]	64.00 [57.00, 70.00]	69.00 [61.00, 73.00]	**0.042**
BMI (kg/m^2^)	23.80 [22.10, 25.90]	23.62 [21.70, 25.78]	24.02 [22.60, 25.95]	0.241
COPD, n (%)	39 (23.5)	21 (24.7)	18 (22.2)	0.846
Coronary heart disease, n (%)	29 (17.5)	9 (10.6)	20 (24.7)	**0.029**
Hypertension, n (%)	34 (20.5)	14 (16.5)	20 (24.7)	0.263
Diabetes, n (%)	28 (16.9)	12 (14.1)	16 (19.8)	0.446
Cancer, n (%)	14 (8.4)	7 (8.2)	7 (8.6)	1.000
GERD, n (%)	6 (3.6)	2 (2.4)	4 (4.9)	0.634
Oxygenation index	357.00 [276.25, 407.00]	376.00 [279.00, 414.00]	357.00 [262.00, 395.00]	0.231
WBC count (10^9^/L)	7.10 [5.80, 8.74]	7.60 [6.20, 9.10]	6.50 [5.60, 8.10]	0.058
Neutrophil (%)	61.10 [54.97, 68.92]	60.00 [55.40, 66.70]	61.60 [53.80, 70.40]	0.411
Lymphocyte (%)	25.80 [19.18, 32.08]	26.60 [21.40, 32.40]	24.00 [18.00, 32.00]	0.131
Monocyte (%)	8.05 [6.50, 9.70]	8.00 [6.40, 9.40]	8.10 [6.60, 10.40]	0.096
Albumin (g/L)	37.45 [34.10, 40.25]	38.90 [35.10, 41.60]	36.50 [33.00, 38.60]	**0.001**
Creatinine (μmol/L)	77.35 [67.10, 88.00]	77.50 [67.00, 89.00]	77.00 [67.60, 86.00]	0.842
Uric acid (μmol/L)	331.00 [270.50, 385.75]	334.00 [281.00, 389.00]	328.00 [256.30, 380.00]	0.234
VCmax (L)	2.41 [1.99, 3.05]	2.51 [2.06, 3.16]	2.37 [1.91, 2.91]	0.160
Vcmax (pred %)	79.50 [63.20, 91.97]	82.30 [62.00, 92.08]	78.20 [66.20, 91.30]	0.957
FVC (L)	2.41 [1.99, 3.03]	2.51 [2.04, 3.16]	2.35 [1.91, 2.83]	0.105
FVC (pred %)	81.20 [65.19, 94.80]	84.90 [63.69, 95.67]	80.20 [68.40, 94.10]	0.963
FEV1 (L)	1.96 [1.65, 2.37]	2.04 [1.73, 2.41]	1.87 [1.55, 2.32]	0.066
FEV1 (pred %)	82.42 [66.21, 97.80]	82.20 [65.60, 98.20]	82.55 [70.00, 97.50]	0.748
FEV1/FVC (%)	81.72 [75.78, 85.93]	82.20 [76.00, 85.88]	81.11 [75.70, 86.44]	0.759
DLCO/VA ≤ 56.03 (pred %)	61 (36.7)	30(35.5)	31 (38.3)	0.813
PMA (cm^2^)	34.14 [27.91, 39.92]	34.67 [27.90, 39.25]	33.14 [27.95, 40.25]	0.947
PMD (HU)	38.46 [34.56, 43.96]	37.94 [34.44, 43.80]	39.14 [35.47, 44.29]	0.759
SATA (cm^2^)	30.52 [20.19, 37.90]	30.41 [20.09, 37.57]	30.60 [21.45, 37.93]	0.585
SATD (HU)	− 97.69 [− 106.41, − 90.92]	98.63 [− 109.47, − 91.39]	− 97.28 [− 103.77, − 90.90]	0.166
PAT (cm^3^)	107.79 [87.21, 149.90]	108.60 [80.25, 154.60]	107.23 [90.49, 136.64]	0.782
Treatment, n (%)				0.393
N-acetylcysteine	59 (35.5)	26 (30.6)	33 (40.7)	
Pirfenidone	83 (50.0)	48 (56.5)	35 (43.2)	
Nintedanib	6 (3.6)	3 (3.5)	3 (3.7)	
Glucocorticoid	18 (10.8)	8 (9.4)	10 (12.3)	

The data were expressed as median [Q1, Q3] or n (%)

The bold p value means p < 0.05 with significantly statistical difference

### Prognostic influencing factors of IPF

Ten significant factors were identified by Cox univariate analysis (Additional file 1: Fig. S1), including two peripheral blood laboratory indices, six lung function indices and two imaging indices. Of these, the neutrophil percentage and lymphocyte percentage were highly correlated, and the lung ventilation indices (VCmax, FVC, FEV1, and FEV1/FVC) were also highly correlated (Spearman correlation coefficient > 0.6). Only one of the similar indices was included. There were five independent predictors of TFS of IPF patients through Cox multivariate regression analysis: neutrophil percentage > 68.3% (HR 5.11, 95% CI 2.57–10.20, p < 0.001), VCmax < 90.5 pred% (HR 2.38, 95% CI 1.04–5.56, p = 0.041), DLCO/VA ≤ 56.03 pred% (HR 3.57, 95% CI 1.85–7.14, p < 0.001), PMD ≤ 36.24 HU (HR 2.63, 95% CI 1.39–5.00, p = 0.003), and PAT > 94.91 cm^3^ (HR 2.44, 95% CI 1.10–5.45, p = 0.029). All five binary variables satisfied the proportional hazards assumption (p < 0.05 by log-rank test) (Additional file 1: Fig. S2).

**Fig. 3:**
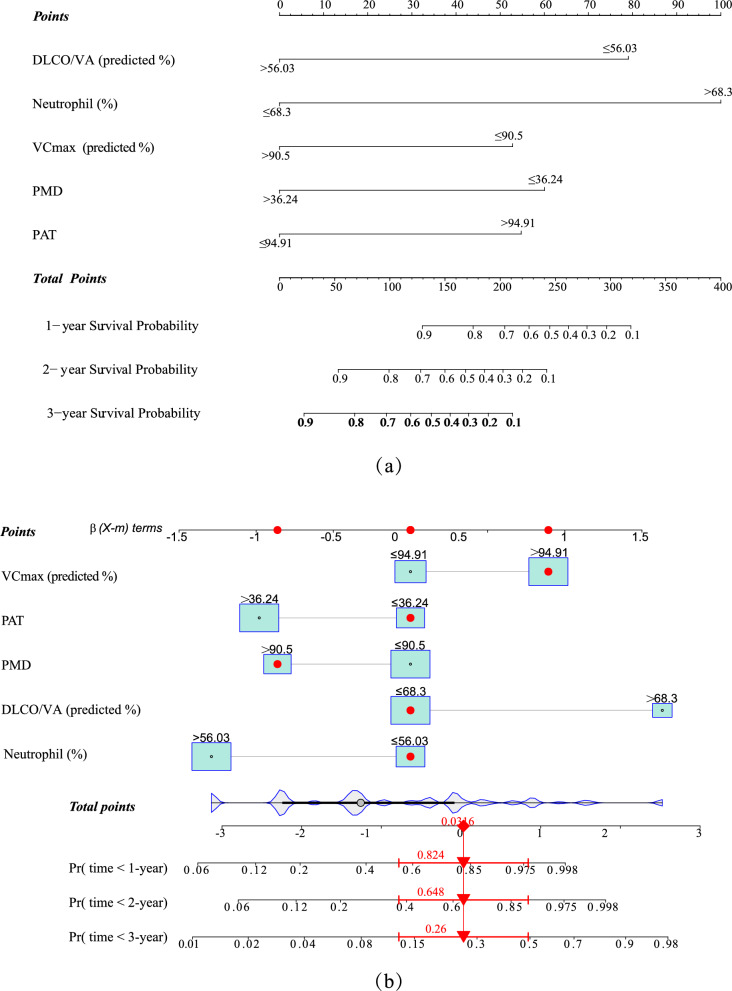
BRP prognostic model for IPF. **a** BRP model. For example, a newly diagnosed IPF patient received the following scores for each factor: DLCO/VA (pred %) ≤ 56.03, score of 79; neutrophil percentage ≤ 68.3%, score of 0; VCmax > 90.5 (pred %), score of 0; PMD ≤ 36.24 HU, score of 60; PAT > 94.91 cm^3^, score of 55. The total BRP score for this patient is 194. **b** The corresponding one-year, two-year, and three-year mortality rates for this patient, based on the total BRP score, are 26.0%, 64.8%, and 82.4%, respectively. These findings suggest that considering lung transplantation within the second to third year after the initial diagnosis could be a beneficial option to avoid the high mortality risk in the third year

**Fig. 4:**
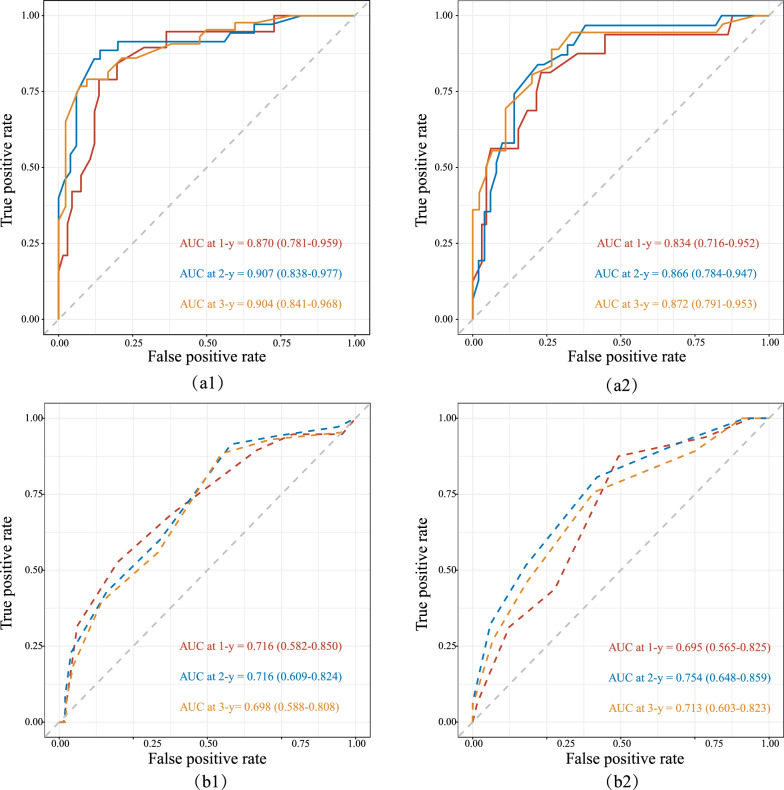
Time-ROC. The BRP scores for predicting 1-year, 2-year, and 3-year TFS in the training set (**a1**) and validation set (**a2**) were shown, along with the GAP scores (dashed line) for the same predictions in the training set (**b1**) and validation set (**b2**)

The variable selection for model construction was performed by stepwise regression. The five significant variables in the Cox analysis were sequentially introduced and not eliminated. Ultimately, an optimal variable set was obtained to construct the prognostic model, named the “blood routine examination-radiology-pulmonary function (BRP) model” (Fig. 3). The AUCs of the ROC curve for the BRP model for 1 year, 2 years, and 3 years in the training set for IPF were 0.870 (95% CI 0.781, 0.959), 0.907 (0.838, 0.977), and 0.904 (0.841, 0.968), respectively. The validation set confirmed the good predictive performance of the BRP model, with AUC values of 0.834 (0.716, 0.952), 0.866 (0.784, 0.947), and 0.872 (0.791, 0.953) for 1-year, 2-year, and 3-year predictions, respectively (Fig. 4). The AUC and 95% CI of the GAP model are also displayed in Fig. 4. The AUC values of the BRP model for 1-year, 2-year, and 3-year predictions in the training set were significantly higher than those of the GAP model (1 year: p-adjust = 0.034, 2 years: p-adjust = 0.009, 3 years: p-adjust = 0.001). In the validation set, there was no statistically significant difference in the AUC values between the two models for the 1-year and 2-year predictions (1 year: p-adjust = 0.124, 2 years: p-adjust = 0.135), but the AUC of the BRP model was significantly higher than that of the GAP model for the 3-year prediction (p-adjust = 0.018). These findings suggest that the BRP model has higher overall accuracy.

**Fig. 5:**
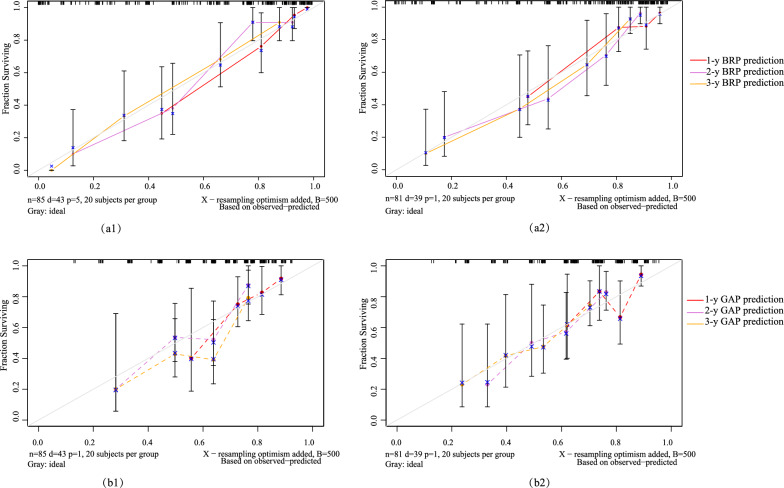
Calibration curves. The BRP model in the training set (**a1**) and validation set (**a2**) and the GAP model in the training set (**b1**) and validation set (**b2**) were shown. The X-axis represents the predicted IPF survival probabilities based on the prediction model, while the Y-axis represented the actual outcomes from the follow-up. The diagonal gray line represented the perfect prediction of an ideal model. The solid lines in red (1-year), pink (2-year), and yellow (3-year) represented the performance of the prediction model

Figure 5 displays the calibration plots and bootstrap resampling results (n = 500) for the training and validation sets, which demonstrate that the BRP model’s predicted outcomes closely align with the actual follow-up results, indicating stable and generalizable predictive performance. The calculation of the IDI suggests that the BRP model has improved predictive ability compared to the GAP model, with improvements of 34.9% (95% CI 20.6%, 49.3%) in the training set and 28.2% (5.9%, 44.6%) in the validation set.

**Fig. 6:**
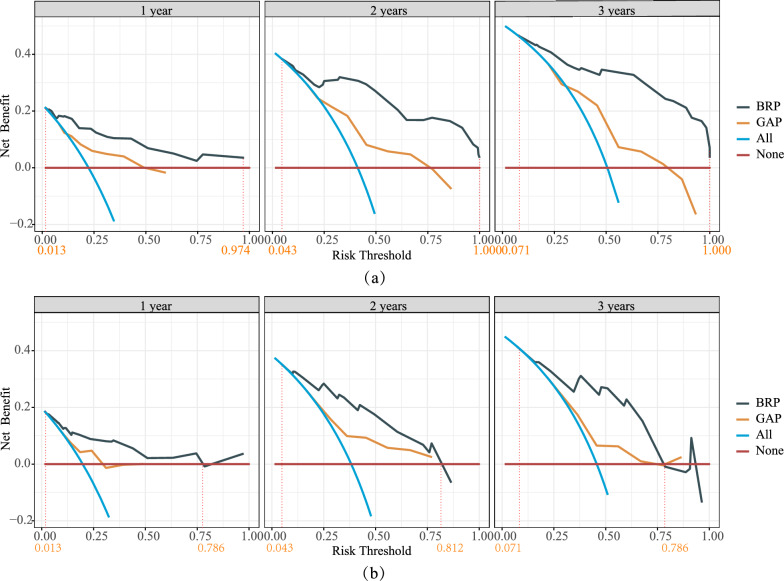
The DCA of the BRP and GAP models in the training set (**a**) and validation set (**b**). The Y-axis represented net benefit, and the X-axis represented the risk thresholds for IPF patients at respective time points

The clinical utility of the two models was evaluated through DCA (Fig. 6). Using the BRP model provided greater clinical benefits compared to using the GAP model in a wider range of applicable risk thresholds. In the validation set, the risk threshold ranges for the BRP model at 1, 2, and 3 years were 1.3% to 78.6%, 4.3% to 81.2%, and 7.1% to 78.6%, respectively, indicating a broader applicability range and superior clinical practicality.

### Risk-stratified analyses by prediction models in IPF patients

**Fig. 7:**
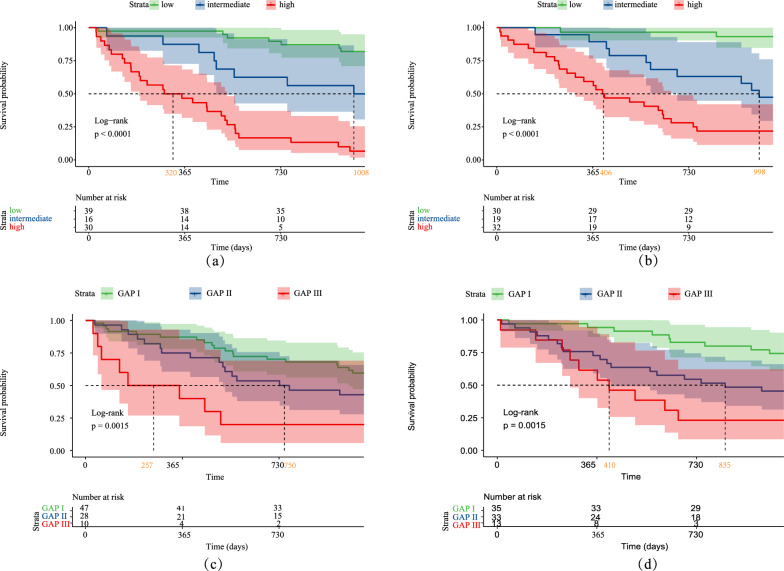
Kaplan–Meier survival analysis. Prognostic risk stratification of the BRP model in the training set (**a1**) and validation set (**a2**), and the GAP index in the training set (**b1**) and validation set (**b2**)

The total scores of the BRP model were calculated for each IPF patient. The patients were divided into three groups, low-risk group, moderate-risk group, and high-risk group, according to their respective BRP scores, using the 25th percentile (115 points) and 75th percentile (187 points) of the total scores in the training set as cutoff values. Statistically significant separation of TFS was observed among different BRP risk groups (Fig. 7a): moderate vs. low (training set: HR 3.58 [95% CI 1.30–9.88], p = 0.014; validation set: 5.02 [1.57–16.03], p = 0.007) and high vs. low (training set: 13.91 [5.92–32.67], p < 0.001; validation set: 12.45 [4.29–36.17], p < 0.001). The patients in the moderate-risk and high-risk groups had significantly shorter survival than those in the low-risk group. The patients in the training and validation sets were stratified into different risk groups according to the established GAP index classification [5] (Fig. 7b). The differences in TFS among the different risk groups are presented in Table 2. In the training set, there was no significant difference in TFS between GAP index Ι and GAP index II (p = 0.143).

**Table 2:** Prognostic differences among different risk stratification of the BRP model and the GAP index

	The training set				The validation set			
	TFS (days)	HR	95% CI	p-value	TFS (days)	HR	95% CI	p-value
BRP moderate-risk^a^	1008	3.58	1.30, 9.88	**0.014**	998	5.02	1.57, 16.03	**0.007**
BRP moderate-risk^a^	> 1100	13.91	5.92, 32.67	** < 0.001**	> 1100	12.45	4.29, 36.17	** < 0.001**
GAP index II^b^	750	1.64	0.84, 3.20	0.143	835	2.73	1.27, 5.88	**0.010**
GAP index III^b^	> 1100	4.24	1.84, 9.78	** < 0.001**	> 1100	4.96	2.04, 12.04	** < 0.001**

The bold p value means p < 0.05 with significantly statistical difference

^a^ means the comparison with the low-risk group of BRP

^b^ means the comparison with GAP index Ι

### Histopathology and TGF-β1 protein expression in different risk groups

Additional file 1: Fig. S3a shows H and E staining results of lung tissues from controls and patients with IPF. Biopsies from the BRP high-risk patients display end-stage fibrosis with mucus-filled honeycomb cysts. Biopsies from BRP low- and intermediate-risk patients demonstrate varying degrees of patchy, peripherally accentuated fibrosis. Additionally, patients with a high-risk stratification (n = 4) according to the BRP model exhibited higher fibrosis scores on histopathology (Additional file 1: Fig. S3b) and higher levels of TGF-β1 protein expression (Additional file 1: Fig. S3c) compared to those in the normal controls (n = 4), low-risk (n = 3), and intermediate-risk groups (n = 3). The results of western blotting analysis also showed an increase in the expression of mature TGF-β1 protein levels in the high-risk group compared to those in the normal controls and low-risk groups (Additional file 1: Fig. S4).

### Analysis of the correlation between serum inflammatory factors and quantitative CT body composition and lung function

**Fig. 8:**
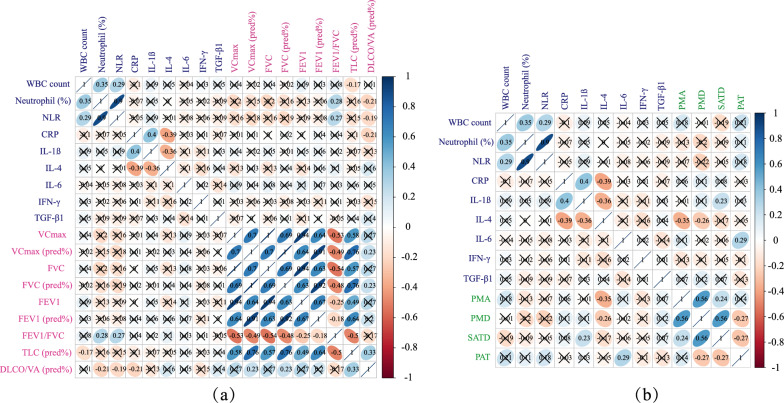
Correlation analysis. **a** Correlation between inflammatory markers (blue) and lung function (pink); **b** correlation between inflammatory markers and body composition (green). Blue presented highly positive correlation coefficients, whereas red presented highly negative correlation coefficients

In Fig. 8a, inflammatory markers showed negative correlations with pulmonary ventilation diffusion function. The percentage of neutrophils was negatively correlated with FEV1/FVC (r = 0.28, p = 0.03), similar to the correlation between the neutrophil-to-lymphocyte ratio (NLR) and FEV1/FVC. Peripheral blood neutrophil percentage, NLR, and serum CRP level were negatively correlated with DLCO/VA (pred%) (r = − 0.21, p = 0.01; r = − 0.19, p = 0.01; and r = 0.28, p = 0.02, respectively). Figure 8b shows the correlation between inflammatory markers and body composition. Serum IL-1β was positively correlated with SATD (r = 0.23, p = 0.03). Higher levels of the profibrotic factor IL-4 were associated with lower PMA and PMD, indicating more significant muscle atrophy and muscle fat infiltration. Higher IL-6 levels were associated with a larger PAT volume (r = 0.29, p = 0.01). No association was detected between TGF-β1 and body composition or lung function. These findings might suggest the rationale for using noninvasive markers, such as PAT, PMD, and PMA, as indicators of inflammation and fibrosis in IPF patients.

## Discussion

Currently, most radiological studies on IPF focus on imaging changes in the lungs, such as ground-glass opacities, traction bronchiectasis, reticular pattern, and honeycombing. CALIPER software developed by the Mayo Clinic in the United States is widely used [28]. However, due to the diverse and heterogeneous nature of lung fibrosis imaging changes, as well as overlapping and indistinct boundaries and the presence of concomitant infections, quantitatively assessing abnormal lung images poses significant challenges. Despite the rapid development of artificial intelligence and deep learning techniques for quantitative CT analysis of interstitial lung diseases in recent years, there is still no convenient and widely recognized prognostic imaging marker for IPF.

Known factors such as aging, hormonal imbalance, and obesity can cause lipids to accumulate in the visceral organs and surrounding areas rather than subcutaneously. This process is known as “ectopic fat deposition.” In respiratory system diseases, visceral fat has become a new research focus due to its impact on increasing disease susceptibility, elevating the diaphragm, compressing airways, promoting inflammation, inducing lipid toxicity, and remodeling the heart and blood vessels [29]. The fat located around the heart and coronary arteries is generally referred to as PAT and is classified as visceral fat tissue. In 2021, Anderson MR and colleagues revealed the association between progressive interstitial lung diseases and visceral fat measured by CT. Their study demonstrated that for every doubling of PAT volume, the odds of measuring interstitial lung abnormalities (ILAs) on CT increased by 20%, and FVC pred% decreased by 5.5 [30]. Another study also reported that excessive PAT was associated with early lung injury and lower FVC [31]. However, some researchers have observed that in these studies, ILA, lung function, and PAT measurements were not conducted simultaneously, which may have affected the research outcomes [32]. Additionally, this study did not utilize these inflammatory and radiological markers for IPF prognosis analysis.

Our research findings indicate that a higher volume of PAT is an independent predictive factor for worse prognosis in patients with IPF (HR 2.44 [95% CI 1.10, 5.45], p = 0.029), and it is associated with higher serum IL-6 levels (r = 0.29, p = 0.01). The transcriptome and secretome of excessive PAT are believed to differ significantly from other fat depots [33], exhibiting a stronger proinflammatory effect and greater lipotoxicity. The current understanding suggests that proinflammatory factors associated with PAT (such as CRP, IL-6, MCP-1, IL-1, and TNF-α mRNA and proteins) can be released into the adjacent pulmonary circulation through blood flow, resulting in lung injury [34]. Elevated levels of IL-6 (> 25.20 pg/mL) have been identified as an independent risk factor for acute exacerbation (OR 1.014 [95% CI 1.001–1.027], p = 0.036) and death (OR 1.007 [95% CI 1.001–1.014], p = 0.018) in patients with interstitial lung disease [35]. High levels of IL-6 have been found in various chronic fibrotic diseases, and IL-6 activates the JAK/STAT3 pathway, promoting fibroblast-to-myofibroblast transformation and collagen deposition in the lungs [36]. In addition to serving as a proinflammatory and endocrine organ, PAT is also associated with functional impairments of the circulatory system. Excessive PAT directly adheres to the surface of the myocardium, exerting constraints on the heart. A study measuring PAT in nearly 7,000 individuals without cardiovascular diseases using CT scans and following up for 17 years showed an increased risk of heart failure associated with excess PAT [35]. The increase in PAT volume is related to more severe hemodynamic disturbances during rest and exercise, including impaired cardiac diastolic function, elevated cardiac filling pressures, and pulmonary hypertension, ultimately leading to reduced cardiopulmonary exercise tolerance in patients [35]. In patients with IPF, this manifests as poor results or even an inability to complete the 6-min walk test (6MWT), which has been proven to be a powerful predictor of high mortality in IPF [6]. However, because visceral obesity is driven by aging, metabolism, inflammation, and dietary factors, PAT may be a modifiable risk factor for IPF, warranting further research on targeted interventions for PAT.

Our study also found that IPF patients with PMD ≤ 36.24 HU had a higher risk of mortality than patients with PMD > 36.24 HU (HR 2.63 [95% CI 1.39, 5.00], p = 0.003). Although PMD showed a positive correlation with PMA (r = 0.56, p = 0.03), there was no statistically significant difference in TFS (HR 0.97, 95% CI 0.93, 1.01, p = 0.084) between patients with high or low PMA in our study. Previous studies have reported a few findings on PMA in IPF [37–39]. However, it is currently difficult to unify the different muscle groups in different planes (such as the 4th and 12th thoracic vertebra; pectoralis major, intercostal muscles, erector spinae, etc.), and the threshold for severe skeletal muscle loss and the extent of its impact on IPF prognosis remains unclear. Therefore, further research is needed [40]. To our knowledge, this is the first study to investigate PMD in IPF and construct an IPF survival prediction model using PMD as the independent variable. In our study, PMD (which reflects both fat infiltration and muscle atrophy) seemed to be a better prognostic indicator than PMA (which only reflects muscle atrophy) was in previous studies. Recent studies have suggested that the deposition of skeletal muscle fat occurs earlier than the reduction in skeletal muscle volume [41]. Lipids and their derivatives accumulate within and between muscle cells, inducing mitochondrial dysfunction, impairing fatty acid β-oxidation, and enhancing reactive oxygen species production, leading to lipotoxicity, insulin resistance, and inflammation and subsequently increasing the risk of systemic muscle loss or even cachexia [42]. This vicious cycle ultimately determines the loss of skeletal muscle quality and strength [43]. Respiratory muscle atrophy may lead to respiratory muscle weakness and dyscoordination, also reflecting the nutritional status of patients, thereby affecting different survival outcomes in respiratory diseases. This view has been previously validated in COPD, asthma [44], and lung cancer, and it was confirmed in IPF for the first time in our study. Our results also emphasize the correlation between profibrotic markers and visceral fat. IL-4 has been reported to activate M2 macrophages and has fibrogenic properties [45]. Our study results suggest that higher serum IL-4 levels are associated with lower PMA and PMD. As an acute-phase protein of inflammation, CRP was not found to be correlated with lung function, skeletal muscle, or PAT in this study. It should be noted that while TGF-β1 is a key factor involved in fibrotic pathways, the use of serum TGF-β1 for predicting the prognosis of patients with IPF was limited in our study. This result was consistent with several previous prognostic studies of patients with IPF [46–48].

In addition to radiological markers, our study also found that IPF patients with a peripheral blood neutrophil percentage greater than 68.3% had a 5.11 times higher risk of poor prognosis than patients with a percentage ≤ 68.3% (95% CI 2.57, 10.20, p < 0.001). Furthermore, the peripheral blood neutrophil percentage and NLR are negatively correlated with pulmonary ventilation and diffusion function. Our study results, consistent with previous research [49, 50], indicate a close relationship between inflammation-related blood cell counts and the severity of IPF. We further integrated the neutrophil percentage into clinical decision-making for IPF. In this study, DLCO/VA pred% was selected as a substitute for DLCO pred% because there is evidence that in IPF patients, gas exchange is more closely related to DLCO/VA than DLCO [51]. Lung transplantation is the final treatment option for severe progression of IPF disease. Without transplantation, it can be expected that patients will experience death in the short term. Therefore, in this study, we selected all-cause mortality or lung transplantation as the endpoint for follow-up.

This study has some limitations. First, although it was a multicenter study, the number of included patients was relatively small. Second, the serum levels of inflammatory factors we measured reflect the overall effect from various cell types and tissues, so the levels of these inflammatory factors in lung tissue remain unknown. Finally, while we excluded patients with acute infections and obvious inflammation, chronic inflammation can have causes other than excess adiposity in IPF. However, we believe that CT-based quantitative body composition is a more stable and simpler indicator than variable lung imaging in IPF patients.

## Conclusions

In summary, we established a novel prognostic model for predicting TFS in IPF patients by integrating routine clinical data. The differences in pathological biopsies among the BRP risk stratifications highlight the reliability of this model, which has clinical utility that can be validated in future studies across larger IPF cohorts. Implementing the BRP model could effectively assist physicians in the early identification of IPF patients with a potentially fatal disease course and in prioritizing patients for lung transplantation. Moreover, the relationship among PAT, PMD, inflammation, and fibrosis undoubtedly warrants further investigation, as it may uncover new targets for IPF treatments by elucidating the proinflammatory pathways related to body composition. Overall, our study provides a convenient prognostic model and innovative insight that may benefit the clinical management of IPF.

### Supplementary Information

**Additional file 1:** **Figure S1.** The forest plot of the Cox univariate and multivariate analyses for TFS in IPF patients. “*” indicates p < 0.05, and “**” indicates p < 0.001. HR represents Hazard Ratio, and 95% CI represents the 95% confidence interval. **Figure S2.** The Kaplan–Meier survival curves for statistically significant variables in COX multivariate analyze. **a** The survival difference based on the high or low proportion of neutrophils, **b** based on PAT (Pericardial adipose tissue), **c** based on PMD (pectoralis muscle radiodensity), **d** based on DLCO/VA (diffusing capacity of the lungs for carbon monoxide/alveolar ventilation), and **e** based on VCmax pred% (percentage of predicted vital capacity). **Figure S3.** Representative images of H and E staining (**a**), Masson’s trichrome staining (**b**) and immunohistochemistry for TGF-β1 (**c**) of lung tissues from normal controls and different BRP risk groups of patients with IPF. “*” indicates p < 0.05, and “**” indicates p < 0.001. **Figure S4.** Western blotting (**a**) and quantitative analysis of TGF-β1 (**b**, **c**) in lung tissues from normal controls and different BRP risk groups of patients with IPF. “*” indicates p < 0.05.

## Data Availability

The datasets used and/or analyzed during the current study are available from the corresponding author on reasonable request.

## Abbreviations

AUC: Area under the receiver operating characteristic curve
BMI: Body mass index
COPD: Chronic obstructive pulmonary disease
CRP: C-reactive protein
CT: Computed tomography scan
DCA: Decision curve analysis
DLCO: Diffusing capacity of the lung for carbon monoxide
DLCO/VA: DLCO/alveolar ventilation
FEV1/FVC: 1St second and forced vital capacity ratio
FVC: Forced vital capacity
GAP: Gender-age-physiology variables
HR: Hazard ratio
HRCT: High-resolution CT
IDI: Integrated discrimination improvement
IFN-γ: Interferon-γ
IL-1β: Interleukin-1β
IL-4: Interleukin- 4
IL-6: Interleukin- 6
IPF: Idiopathic pulmonary fibrosis
PAT: Paracardial adipose tissue
PMA: Pectoralis muscle area
PMD: Pectoralis muscle radiodensity
Pred %: Predicted percentage
SAT: Subcutaneous adipose tissue
SATA: Subcutaneous adipose tissue area
SATD: Subcutaneous adipose tissue density
TFS: Transplant-free survival
time-ROC curve: Time-dependent receiver operating characteristic curve
TNF-α: Tumor necrosis factor-alpha
VAT: Visceral adipose tissue
VCmax: Maximum vital capacity
95% CI: 95% Confidence interval
6MWT: 6-Minute walk test
