# Supplementary material for: Establishment and application of the BRP prognosis model for idiopathic pulmonary fibrosis
Source: J Transl Med. 2023 Nov 11;21:805. doi: 10.1186/s12967-023-04668-5 (PMC10638707; doi:10.1186/s12967-023-04668-5)
Supplement: Supplementary file 1 — Additional file 1: Figure S1. The forest plot of the Cox univariate and multivariate analyses for TFS in IPF patients. “*” indicates p < 0.05, and “**” indicates p < 0.001. HR represents Hazard Ratio, and 95% CI represents the 95% confidence interval. Figure S2. The Kaplan–Meier survival curves for statistically significant variables in COX multivariate analyze. a The survival difference based on the high or low proportion of neutrophils, b based on PAT (Pericardial adipose tissue), c based on PMD (pectoralis muscle radiodensity), d based on DLCO/VA (diffusing capacity of the lungs for carbon monoxide/alveolar ventilation), and e based on VCmax pred% (percentage of predicted vital capacity). Figure S3. Representative images of H and E staining (a), Masson’s trichrome staining (b) and immunohistochemistry for TGF-β1 (c) of lung tissues from normal controls and different BRP risk groups of patients with IPF. “*” indicates p < 0.05, and “**” indicates p < 0.001. Figure S4. Western blotting (a) and quantitative analysis of TGF-β1 (b, c) in lung tissues from normal controls and different BRP risk groups of patients with IPF. “*” indicates p < 0.05. [file 12967_2023_4668_MOESM1_ESM.docx]

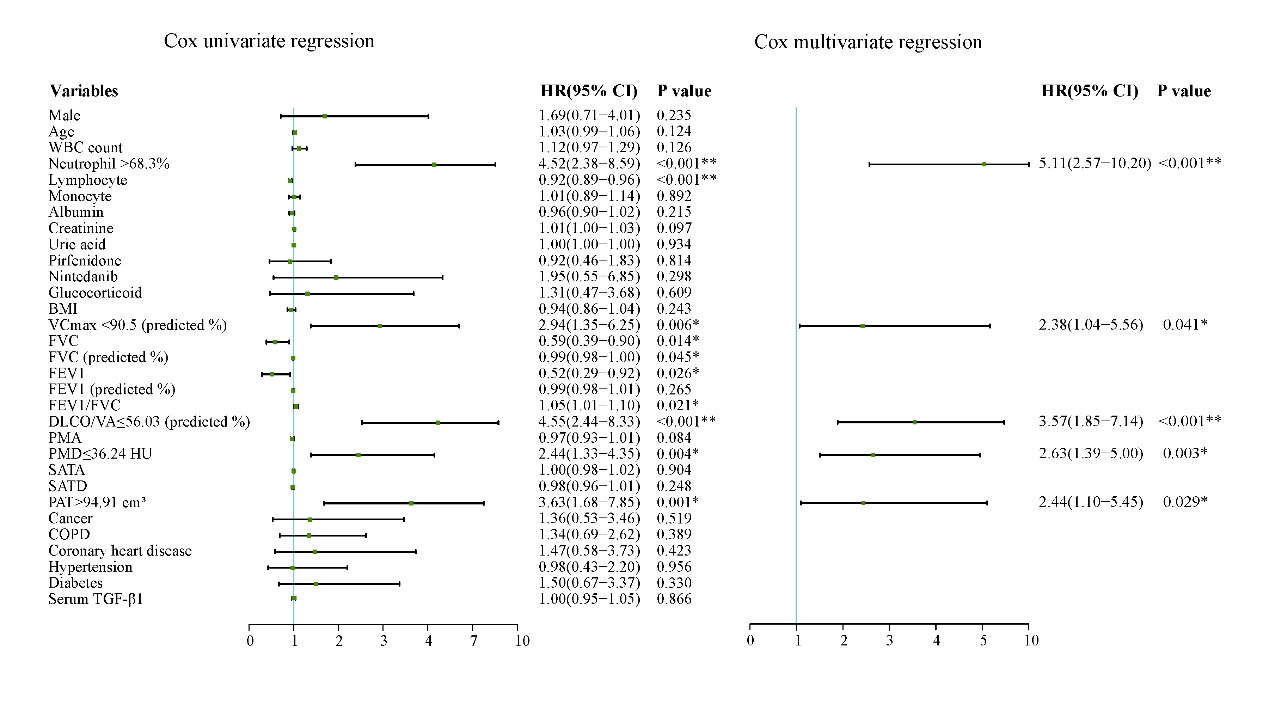
 **Fig. S1** The forest plot of the Cox univariate and multivariate analyses for TFS in IPF patients. “*” indicates p<0.05, and “**” indicates p<0.001. HR represents Hazard Ratio, and 95% CI represents the 95% confidence interval.


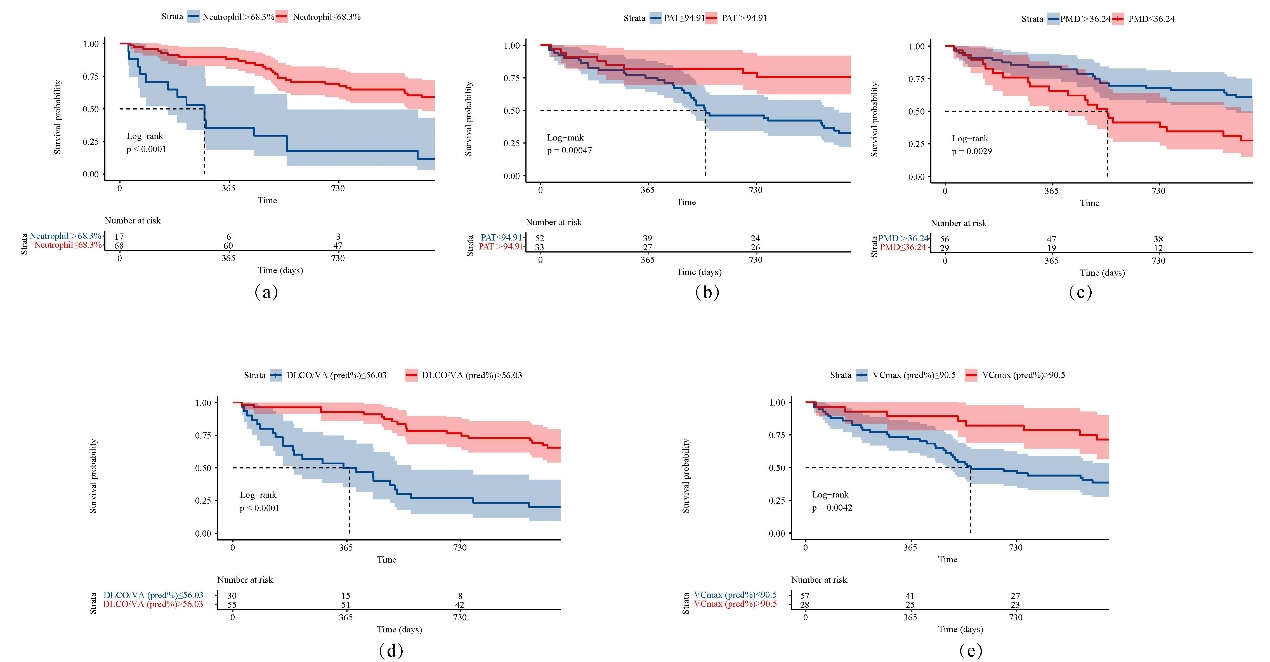


**Fig. S2** The Kaplan-Meier survival curves for statistically significant variables in COX multivariate analyze. (a) The survival difference based on the high or low proportion of neutrophils, (b) based on PAT (Pericardial adipose tissue), (c) based on PMD (pectoralis muscle radiodensity), (d) based on DLCO/VA (diffusing capacity of the lungs for carbon monoxide/alveolar ventilation), and (e) based on VCmax pred% (percentage of predicted vital capacity).


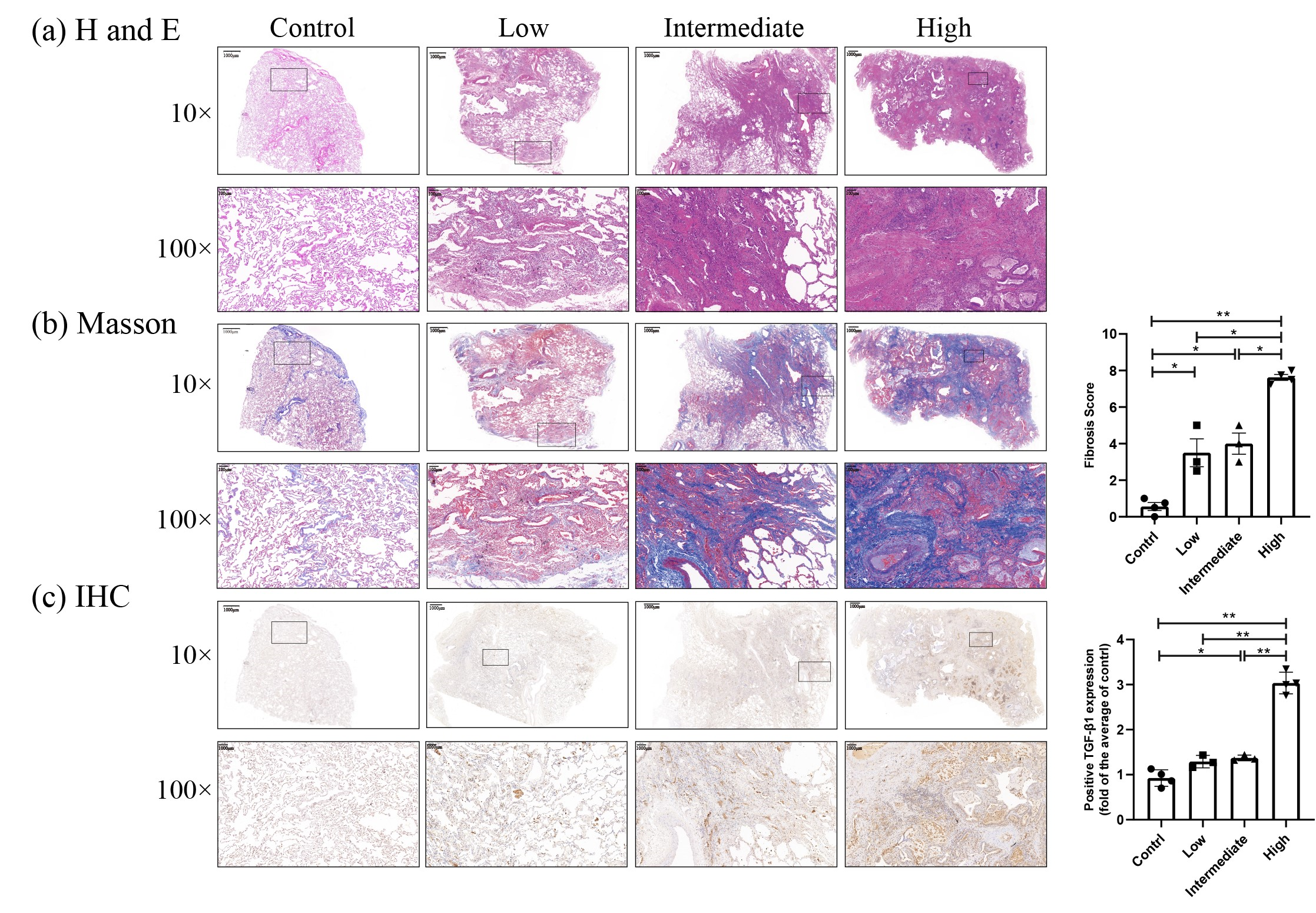


**Fig. S3** Representative images of H and E staining (a), Masson’s trichrome staining (b) and immunohistochemistry for TGF-β1 (c) of lung tissues from normal controls and different BRP risk groups of patients with IPF. “*” indicates p<0.05, and “**” indicates p<0.001.


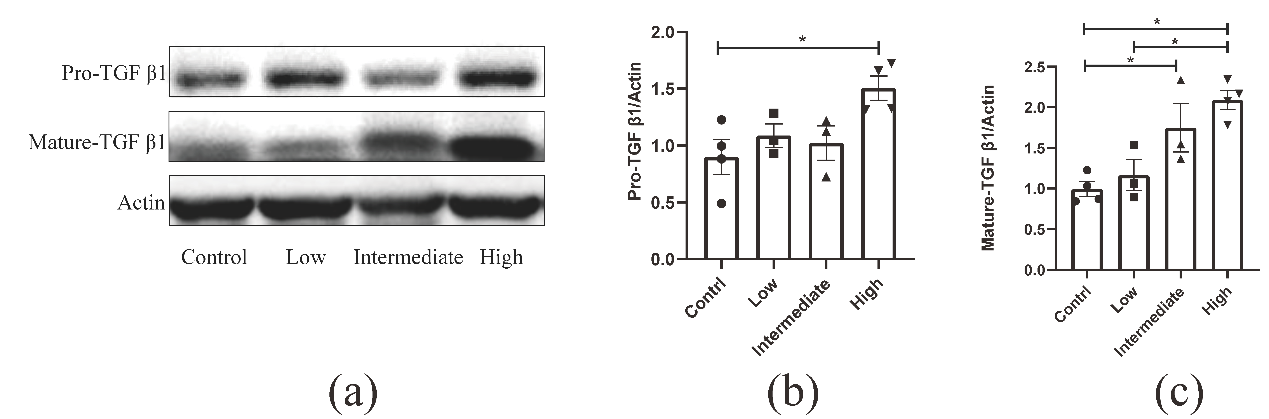


**Fig. S4** Western blotting (a) and quantitative analysis of TGF-β1 (b, c) in lung tissues from normal controls and different BRP risk groups of patients with IPF. “*” indicates p<0.05.
